# Supplementary material for: HER2 Amplification Level Predicts Pathological Complete Response in the Neoadjuvant Setting of HER2-Overexpressing Breast Cancer: A Meta-Analysis and Systematic Review
Source: Int J Mol Sci. 2023 Feb 10;24(4):3590. doi: 10.3390/ijms24043590 (PMC9960382; doi:10.3390/ijms24043590)
Supplement: Supplementary file 1 [file ijms-24-03590-s001.zip › ijms-2053358-supplementary.pdf]

Supplementary Figure Legends

Supplementary Figure 1: Funnel plot for pathological complete response across studies

excluding Greenwell et al. P-value for publication bias = 0.06

Supplementary Figure 2: Risk of bias assessments for 3 randomized clinical trials.

Supplementary Figure 3: Risk of bias assessments for 5 observational studies

Supplementary Figure 4: Risk of bias assessment for 1 single-arm trial.

Supplementary Figure 5: Forest plot for pathological complete response according to the

HER2 interval (tertiles)

Supplementary Figure 6: Forest plot for pathological complete response according to the

HER2 interval (quartiles) and excluding the class with a HER2/CEP17 ratio ≤ 2

Supplementary Figure

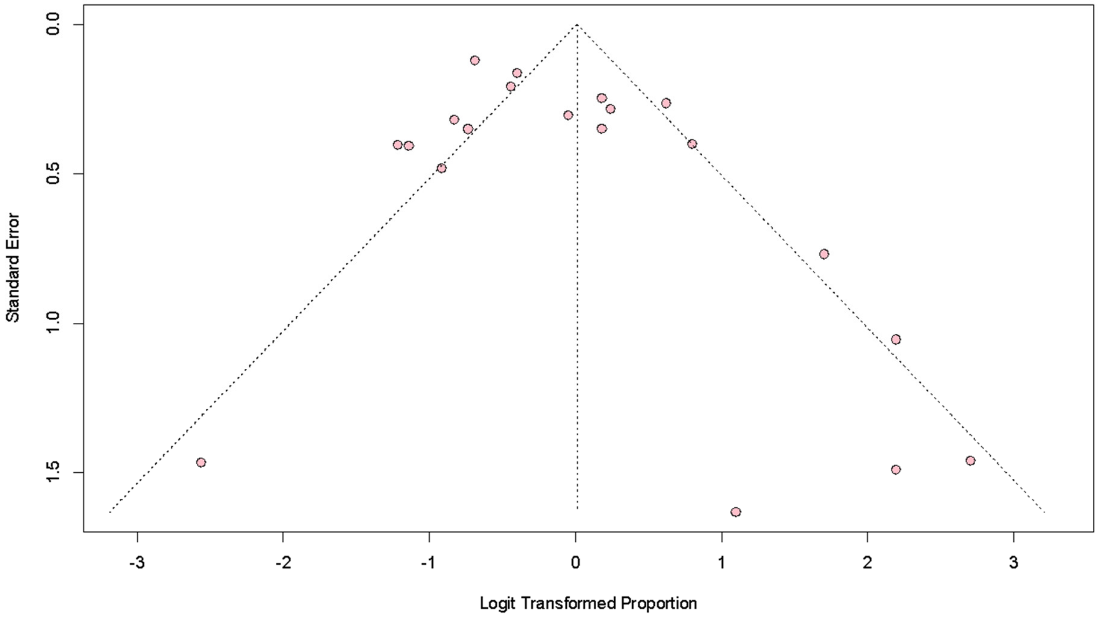

Supplementary Figure 1

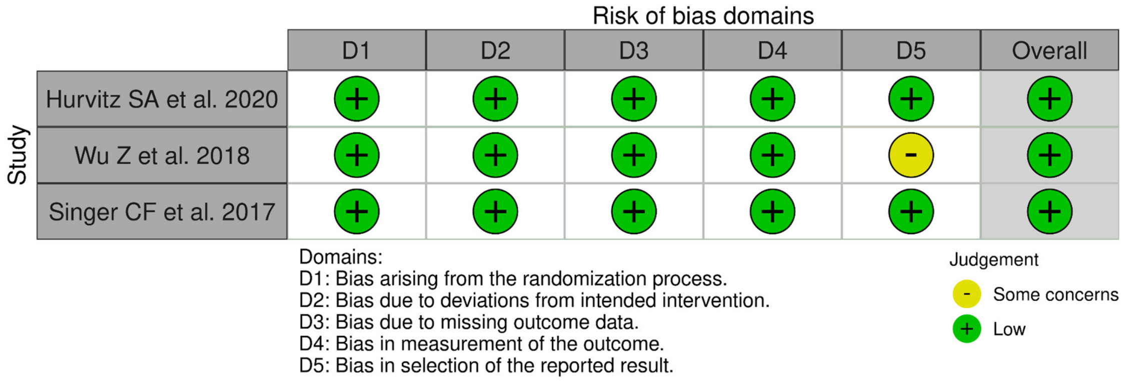

Supplementary Figure 2

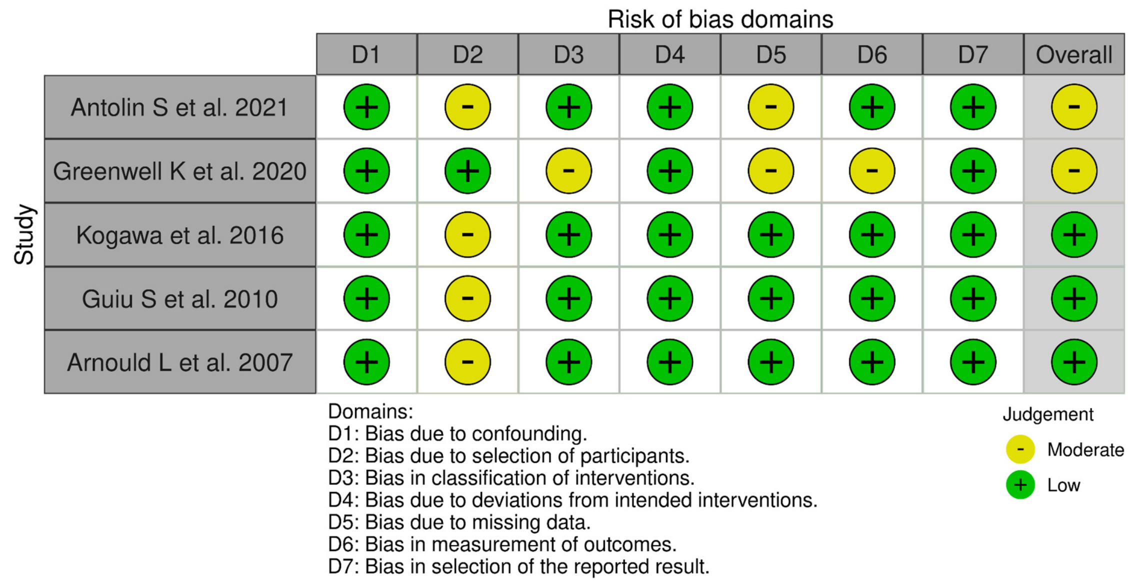

Supplementary Figure 3

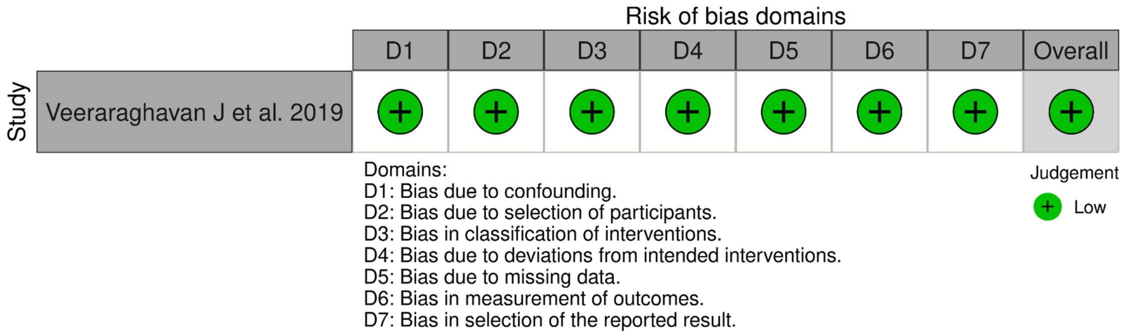

Supplementary Figure 4

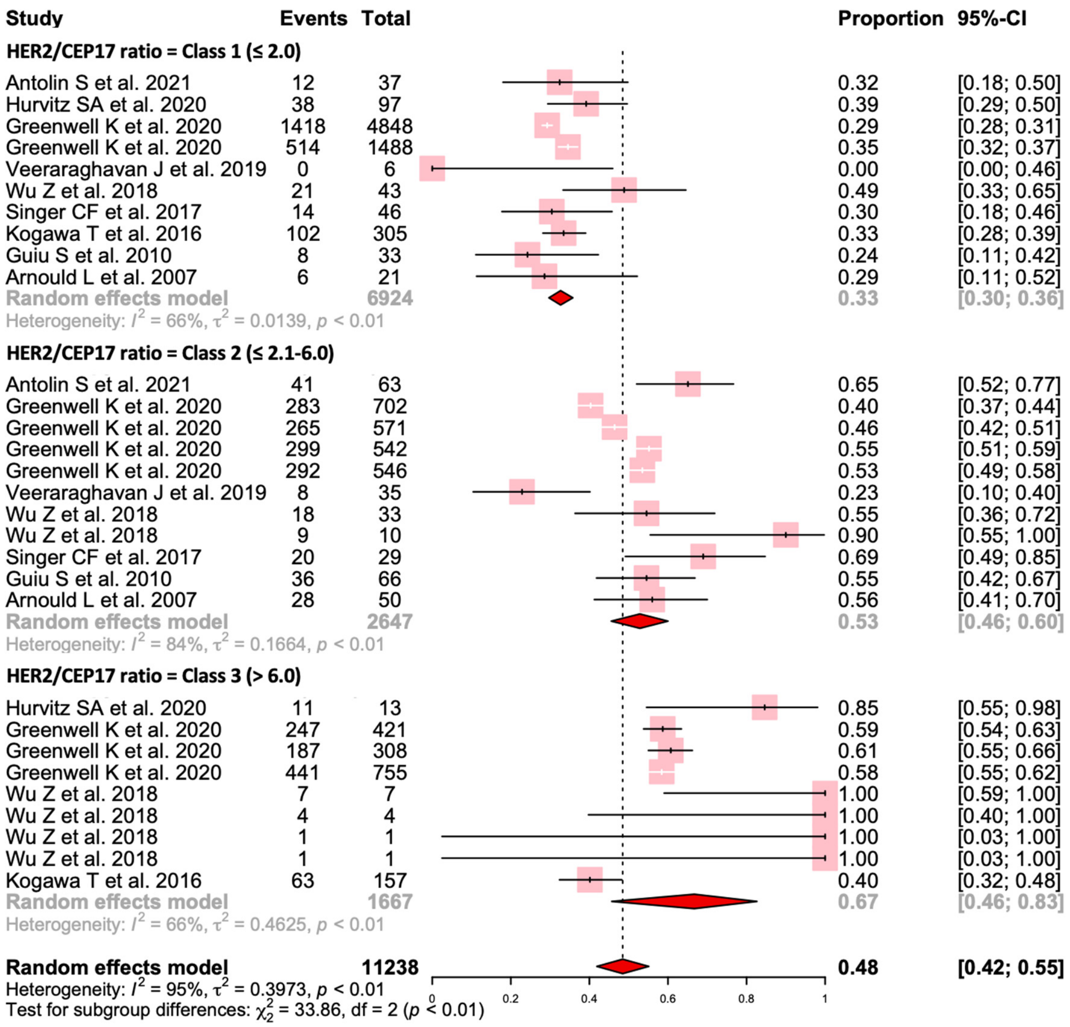

Supplementary Figure 5

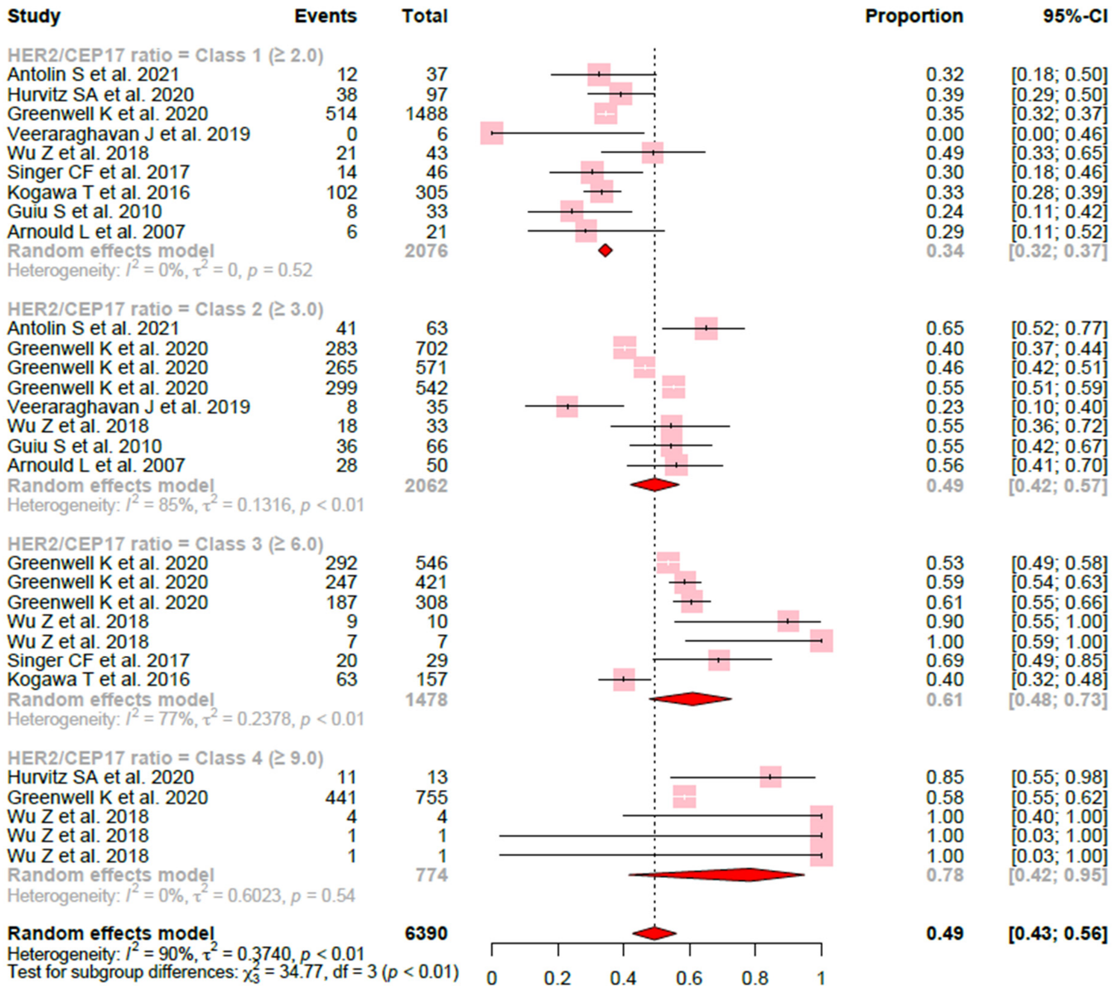

Supplementary Figure 6
